# Resistance to Second-Line Antituberculosis Drugs and Delay in Drug Susceptibility Testing among Multidrug-Resistant Tuberculosis Patients in Shanghai

**DOI:** 10.1155/2016/2628913

**Published:** 2016-08-29

**Authors:** Yong Chen, Zhengan Yuan, Xin Shen, Jie Wu, Zheyuan Wu, Biao Xu

**Affiliations:** ^1^Department of Epidemiology, School of Public Health, Fudan University, 138 Dongan Road, Shanghai 200032, China; ^2^Shanghai Municipal Center for Disease Control and Prevention, 1380 West Zhongshan Road, Shanghai 200336, China

## Abstract

*Introduction*. Second-line antituberculosis drugs (SLDs) are used for treating multidrug-resistant tuberculosis (MDR-TB). Prolonged delays before confirming MDR-TB with drug susceptibility testing (DST) could result in transmission of drug-resistant strains and inappropriate use of SLDs, thereby increasing the risk of resistance to SLDs. This study investigated the diagnostic delay in DST and prevalence of baseline SLD resistance in Shanghai and described the distribution of SLD resistance with varied delays to DST.* Methods*. All registered patients from 2011 to 2013 in Shanghai were enrolled. Susceptibility to ofloxacin, amikacin, kanamycin, and capreomycin was tested. Total delay in DST completion was measured from the onset of symptoms to reporting DST results.* Results*. Resistance to SLDs was tested in 217 of the 276 MDR-TB strains, with 118 (54.4%) being resistant to at least one of the four SLDs. The median total delay in DST was 136 days. Patients with delay longer than median days were roughly twice more likely to have resistance to at least one SLD (OR 2.22, 95% CI 1.19–4.11).* Conclusions*. During prolonged delay in DST, primary and acquired resistance to SLDs might occur more frequently. Rapid diagnosis of MDR-TB, improved nosocomial infection controls, and regulated treatment are imperative to prevent SLD resistance.

## 1. Introduction

Multidrug-resistant tuberculosis (MDR-TB) is caused by infection with strains of* Mycobacterium tuberculosis* (*Mtb*) that are resistant to at least isoniazid and rifampicin [1]. Treatment of MDR-TB requires longer course duration and more expensive drugs with higher risk of toxic adverse effects, compared with treatment of drug-susceptible TB [2]. Current guidelines of the World Health Organization (WHO) [3] recommend that MDR-TB should be treated with a multidrug regimen consisting of at least four drugs, including first-line drugs (FLDs) such as ethambutol, streptomycin, and pyrazinamide, as well as second-line drugs (SLDs) such as fluoroquinolones (FQs) and second-line injectable drugs including amikacin, kanamycin, and capreomycin.

Resistance to anti-TB drugs emerges primarily owing to inappropriate treatment regimens, poor adherence to regulated treatment, previous use of anti-TB drugs, and primary infection with drug-resistant strains [4, 5]. Recent studies have suggested that resistance to SLDs has arisen as a new threat [5–7], leading to extensively drug-resistant tuberculosis (XDR-TB), which has been found in 105 countries/regions thus far [8], and even so-called totally drug-resistant tuberculosis [5, 9]. According to the most recent national survey in China, it was estimated that 110,000 patients developed MDR-TB, among which 8,200 were XDR-TB in 2007 [10]. In Shanghai, Li et al. reported that SLD resistance existed among 51.8% MDR-TB patients [11]. In another study, 25.1% MDR-TB patients showed resistance to FQs [12]. With greater resistance to SLDs, it is more difficult to achieve treatment success and to control the spread of drug-resistant TB.

To determine MDR-TB and initiate treatment promptly, a timely drug susceptibility testing (DST) is essential. To date, adequate laboratory capacities for rapid DST are yet to be established globally and are insufficient in high TB burden settings including China, one of the countries having heavy MDR-TB burdens [8]. A recent study reported a median delay of 102 days before getting the DST results in Shandong Province [13]. It also mentioned that misuse of SLD is common before MDR-TB diagnosis and during the routine practice of TB treatment. Given that delayed DST could result in further transmission of drug-resistant strains and inappropriate regimen consisting of SLDs [4, 10], it is important to understand both the delays in DST before MDR-TB diagnosis and the baseline resistance to SLDs, in order to develop appropriate MDR-TB treatment regimen rapidly and strengthen control measures. This study aimed to investigate the diagnostic delay before DST and the prevalence of baseline SLD resistance among MDR-TB patients in Shanghai, one of the most developed cities of China, further, to describe the distribution of SLD resistance among patients with varied delays to DST and its influence factors.

## 2. Methods

### 2.1. TB Health Care Setting

A MDR-TB control program has been set up in Shanghai since 2011, as a part of the city's comprehensive TB control program. According to the TB control setting in Shanghai, suspected cases of pulmonary TB detected in any health care facilities should be referred to designated TB hospitals, where sputum smear, culture, and chest radiography are routinely performed upon patients arrival. Isolates from positive culture are then sent to the Province Tuberculosis Reference Laboratory at the Shanghai Municipal Centre for Disease Control and Prevention (Shanghai CDC), where strain differentiation and DST for four FLDs (isoniazid, rifampicin, ethambutol, and streptomycin) are routinely performed using the proportion method [14]. Following regulations of the Shanghai MDR-TB control program, patients with* Mtb* strains that are resistant to at least isoniazid and rifampicin should be considered as suspected MDR-TB cases. These patients should be referred to the designated MDR-TB hospitals after DST results being reported. Diagnosis of MDR-TB is given by group consensus including pulmonary specialists and public health experts, based on DST results and clinical features in these hospitals. Confirmed MDR-TB patients will be provided for a regulated 24-month treatment course with the WHO recommended regimens.

### 2.2. Study Design

The study was an observational, retrospective study using data from the TB registry and the MDR-TB management program in Shanghai. All patients with confirmed diagnosis of MDR-TB between April 2011 and December 2013 under the Shanghai MDR-TB control program were enrolled as study participants. The main outcome of this study is delays in DST, as well as baseline resistance to regular SLDs used in Shanghai.

### 2.3. Data Collection

Information of confirmed patients was collected from the TB registry at the Shanghai CDC; this included age, sex, residence, hospital of TB diagnosis, and previous TB history. Dates of onset of symptoms and TB diagnosis, which were recorded by clinical doctors and reported to the TB registry as regulated, were retrieved from the registration database as well. Dates of reporting DST results were also documented in the program.

### 2.4. Drug Susceptibility Tests for SLDs

For the current study, DST for SLDs including ofloxacin (Ofx), amikacin (Am), kanamycin (Km), and capreomycin (Cm) were carried out specifically among those diagnosed MDR-TB patients in the BACTEC MGIT 960 system (Becton, Dickinson and Company, Franklin Lakes, NJ, USA) [15] at the Shanghai CDC and Shanghai Pulmonary Hospital. The tests were performed using drugs (Sigma, Taufkirchen, Germany) at the following concentrations: Ofx (2.0 mg/mL), Am (1.0 mg/mL), Km (2.5 mg/mL), and Cm (2.5 mg/mL). All DST for SLDs were performed by senior technician with WHO certification. Resistance to Am and Km was categorized into one subgroup, owing to the high prevalence of cross-resistance between these drugs.

### 2.5. Definitions

MDR-TB is defined as tuberculosis caused by strains of* Mtb* that are resistant to at least isoniazid and rifampicin. XDR-TB is defined as MDR-TB with additional resistance to Ofx and at least one second-line injectable drug [16]. Total delay in DST completion was defined as the duration from onset of TB symptoms in the most recent disease episode to the date of reporting DST results in laboratories, which was further divided into delay in TB diagnosis (the duration from onset of TB to the date of receiving a TB diagnosis by smear microscopy) and health system delay in DST completion (the duration from TB diagnosis to reporting DST results). Patients were categorized according to WHO criteria [17] and the Chinese MDR-TB treatment guideline [18]. A new patient was defined as a patient who had never been treated for TB. A relapsed patient was defined as a previously treated patient whose last treatment outcome was successful (cured or completed treatment). A chronic patient was defined as a previously treated patient without a successful outcome in the last treatment episode.

### 2.6. Statistical Analysis

Distributions of resistance to SLDs among patients with different demographic and disease profile were compared using the *χ* ^2^ test. The Kruskal-Wallis test was used to evaluate delays of patients with different drug resistance patterns and TB history. Unconditional logistic regression was used to investigate the association between length of delays and occurrences of SLD resistance by estimating odds ratios (ORs) and 95% confidence intervals (CIs). In the multivariate models, diagnostic delays were dichotomized by median days. Stratification analysis was applied with regard to previous TB history and residential status. Statistical tests were two-tailed with significance level set at *α* < 0.05. All statistical analyses were performed using SAS software, version 9.1 (SAS Institute Inc., Cary, NC, USA).

## 3. Results

### 3.1. Characteristics of Study Population

During the study period, a total of 8,922 patients with bacterial-positive pulmonary tuberculosis were registered in Shanghai, among which 287 (3.2%) were confirmed as MDR-TB; none were HIV-positive. Nine patients (3.1%) were excluded owing to a lack of information on diagnostic procedure. Another two (0.7%) were later excluded for infection with* nontuberculous Mycobacteria*. Of the remaining 276 patients (96.2%), 217 (78.6%) had information on resistance to SLDs. Some patients with favorable sputum conversion in the early phase of TB treatment did not receive DST on SLDs. Other reasons for unavailable SLD profiles were lack of baseline sputum specimen, culture contamination, unsuccessful cultures, and failed DST. No statistically significant differences were found regarding demographic characteristics and disease profile between patients with DST results for SLDs and those without (data not shown).

### 3.2. Resistance to Anti-TB SLDs

Among the 217* Mtb* isolates tested, 90 (41.5%) were resistant to Ofx, 37 (17.1%) were resistant to Am/Km, and 44 (20.3%) were resistant to Cm. One hundred and eighteen patients (54.4%) harbored strains resistant to at least one SLD. Additionally, 30 patients (13.8%) were infected with XDR strains.  Table 1 presents the resistance to SLDs among 217 isolates of patients. Resistance to at least one SLD was positively associated with older age (median years, 49.4 versus 39.6, *p* = 0.002). Local residents were more likely to have resistance to Am/Km (*p* = 0.005) and more XDR-TB (*p* = 0.02), compared with domestic migrants. Chronic patients (*p* = 0.01) and those having TB diagnosis in municipal hospitals (*p* = 0.003) had higher possibility of resistance to at least one SLD.

### 3.3. Delay before DST

Table 2 describes the diagnostic delays among patients with different TB history and drug resistance pattern. The median of total delay in DST completion was 136 days (interquartile range (IQR) 88–235 days), while the median days of delay in TB diagnosis and health system delay in DST completion were 33 (IQR 19–56 days) and 92 (IQR 47–192 days), respectively. Chronic patients had longer total and health system delay in DST completion (*p* < 0.001 for both), compared with newly diagnosed cases. Those who were resistant to Ofx and second-line injectable drugs had longer total delay in DST completion (*p* = 0.006 for Ofx resistance and *p* = 0.004 for Am/Km/Cm resistance) and health system delay in DST completion (*p* = 0.03 for Ofx resistance and *p* = 0.002 for Am/Km/Cm resistance). Delay in TB diagnosis did not vary by drug resistance pattern and TB history.

### 3.4. Distribution of SLD Resistance and Delays in DST

In the multivariate regression analysis, the adjusted ORs of having a total delay in DST longer than median days were 2.22 (95% CI: 1.19–4.11) for resistance to at least one SLD and 2.03 (95% CI: 1.05–3.39) for Ofx resistance. In terms of health system delay in DST completion, the ORs were 3.44 (95% CI: 1.79–6.61) for resistance to at least one SLD and 2.59 (95% CI: 1.32–5.09) for Ofx resistance. Delay in TB diagnosis was not significantly associated with SLD resistance (Table 3). The possible link between delays and SLD resistance was further analyzed, stratified by residential status and previous TB history (Table 4). Local patients experiencing longer delays were more likely to have resistance to any SLD (OR 2.35; 95% CI: 1.08–5.11 for total delay in DST; OR 3.93; 95% CI: 1.71–9.07 for health system delay in DST), as well as to Ofx (OR 2.93; 95% CI: 1.28–6.70 for health system delay in DST). A similar distribution was observed among chronic patients for total delay in DST completion (OR 7.14, 95% CI: 1.46–35.00 for resistance to any SLD; OR 4.65, 95% CI: 1.03–21.08 for Ofx resistance). In terms of health system delay in DST completion, both relapsed (OR 5.75; 95% CI: 1.89–17.52 for resistance to any SLD and 5.01; 95% CI: 1.39–18.05 for Ofx resistance) and chronic patients (OR 5.09; 95% CI: 1.01–25.70 for resistance to any SLD and 4.95; 95% CI: 1.12–21.82 for Ofx resistance) had longer delay.

## 4. Discussion

The emergence of XDR-TB has jeopardized global TB control achievements and threatened the accomplishments of the WHO End TB Strategy. Globally, the treatment success rate among patients with MDR-TB varies between 49% and 65% [8]. This could worsen with increased resistance to anti-TB SLDs [19]. In the current study, long delays before the completion of a conventional DST were observed among MDR-TB patients in Shanghai. Meanwhile, baseline resistance to SLDs was found to be prevalent. Since both primary and acquired drug resistance could occur during unnecessary delay before diagnosis, our findings call for more specific interventions to accelerate the diagnosis of MDR-TB.

In this study, it took a median of 33 days for patients to get a microscopy-based TB diagnosis, which is close to other reports in China [20, 21]. Before conventional DST, several additional weeks would be spent on sputum culturing, patient transferring, and waiting in laboratory. These steps created a median health system delay in DST completion of 92 days. Although it was shorter than that in other province in China and other countries, such as India [13, 22], the total delay in DST (over 4 months) was still substantial when we considered the patient's delay all together. Delayed DST using conventional method could increase the risk of continued transmission of drug-resistant TB, progression of the disease, and poor treatment outcome [4, 10]. In practice, patient contacting and group expert consensus are needed to confirm MDR-TB, and the initiation of proper treatment could be further delayed in Shanghai. Given the high burden of MDR-TB in China [8], this diagnostic gap could cripple the current TB control setting, leading to more drug-resistant patients and lower treatment success rate.

Our population-based observational study demonstrated that 54.4% MDR-TB patients in Shanghai had already had resistance to at least one SLD, which was even worse than that in other reports in China (51.8%) [11], India (44.8%) [23], Russia (43.3%) [24], and Poland (30.4%) [25]. SLD-resistant TB arises mainly from direct transmission [4, 10, 26–28]. In this study, SLD resistance seemed to exist more frequently among patients who endured longer diagnostic delay, after being adjusted with demographic and disease profiles. For example, patients with longer total delay in DST were more than twice more likely to have resistance to Ofx. Similar pattern existed in health system delay in DST. The high SLD resistance rate could be partly driven by transmission of drug-resistant strains in the prolonged diagnostic procedure. Before available DST results, repeated visiting to TB hospitals could occur, and strains with resistance beyond MDR might therefore spread [5, 27]. Currently, there are only 3 MDR-TB hospitals in Shanghai, serving a resident population of over 24 million, together with floating patients from all over the country. Within these hospitals, patients with SLD-resistant strains presented to crowded outpatient departments, in which long waiting lines exist due to the limited capacities, thereby increasing the risk of nosocomial transmission [29], particularly when the ventilation was inadequate [30]. We found that patients diagnosed in 3 municipal level TB hospitals presented more SLD resistance than those in 30 district level hospitals (77 cases, 60.6% versus 36 cases, 42.4%, *p* = 0.003), suggesting the risk of nosocomial transmission in these hospitals.

Despite primary transmission, SLD resistance could be independently acquired [31–33]. During initial treatment of TB, irrational and inadequate use of SLDs could be common in China [13]. Therefore, it is possible that MDR strains could evolve to XDR before initiating treatment with SLDs. Given that FQs, which are widely used in China, can lead to both delayed TB treatment [34, 35] and fluoroquinolone resistance [35, 36], previous use of FQs for treating respiratory and other bacterial infections could further increase the risk of acquired Ofx resistance [34, 37], even with short duration of exposure [37].

The potential link between longer delay and SLD resistance was found only among patients with TB history and local residents. In general, previously treated patients had more visiting to health care facilities. Local residents in Shanghai usually have better financial status and medical insurance and therefore have more opportunity to seek health care. Consequently, the cumulative duration and frequency of exposure to SLD-resistant strains in the health care facilities could be more intensive among these patients. As Hu et al. indicated, multiple transmissions of SLD-resistant strains could occur within local TB dispensaries or communities [26]. This finding suggests that previously treated TB patients and local patients might be at higher risk of SLD resistance.

Considering the long diagnostic delay and increased prevalence of SLD-resistant TB in Shanghai, current measures for MDR-TB control have come under challenge. To address this situation, new generation of rapid DST techniques might be the key, especially for relapsed and chronic patients. For example, the GenoType MTBDR*sl* assay can both reduce diagnostic delay of MDR-TB and detect resistance to FQ and injectable drugs simultaneously [38]. Appropriate regimen with SLDs can be timely formed when these methods are introduced. With the financial resources and comprehensive TB control setting in Shanghai, it is possible to provide all TB patients with rapid DST diagnosis at the time of smear microscopy. Meanwhile, nosocomial infection control should be further improved in TB hospitals, and the use of FQs should be more firmly supervised.

As a registry system based, cross-sectional study, this study incurred some limitations. First, we only had one DST profile for SLDs; thus, the time to resistance to SLDs remained unknown. Further study with multiple follow-up DSTs is essential to understand the development of drug-resistant tuberculosis. Second, times of TB hospitals visiting before DST were not available; thus the frequency of potential exposure to drug-resistant strains remained unclear. Third, ofloxacin and other SLDs may have been used during differential diagnosis and previous treatment, but we were unable to collect such details because history of these drugs was not compulsorily recorded. Fourth, recall bias concerning the onset of symptoms might exists, especially among chronic patients. Besides, resistance to other SLDs, such as prothionamide and* p*-aminosalicylic acid, was not evaluated.

## 5. Conclusions

In summary, the average delay days to get DST results among MDR-TB patients in Shanghai could be longer than 136 days. More than 54% MDR-TB patients had already developed resistance to SLDs at the time of MDR-TB diagnosis. SLD resistance was more likely to occur among patients with longer delay in DST, which suggests the increased risk of direct transmission and stepwise evolved acquisition. The rapid diagnosis of MDR-TB should be scaled up in resource available areas to reduce delay in DST. Also, the use of SLDs should be regulated in TB treatment, in line with the nosocomial infection control in TB hospitals.

## Figures and Tables

**Table 1:** Resistance to second-line drugs among MDR-TB patients by demographic characteristics and disease profile.

Variables	Total	Resistance to at least one SLD (*n* = 118)		Resistance to Ofx (*n* = 90)		Resistance to Am/Km (*n* = 37)		Resistant to Cm (*n* = 44)		XDR-TB (*n* = 30)	
	*n*	*n*	%	*n*	%	*n*	%	*n*	%	*n*	%
*Age at diagnosis (years) *											
<30	48	17	35.4	12	25.0	3	6.3	6	12.5	3	6.3
30–60	137	77	56.2	58	43.2	26	19.0	30	21.9	21	15.3
>60	32	24	75.0	20	62.5	8	25.0	8	25.0	6	18.8
*p* value^a^		<0.001		<0.001		0.02		0.14		0.09	

*Sex*											
Male	157	84	53.5	63	40.1	28	17.8	32	20.4	21	13.4
Female	60	34	56.7	27	45.0	9	15.0	12	20.0	9	15.0
*p* value		0.68		0.51		0.62		0.95		0.76	

*Domestic migrant*											
No	131	77	58.8	61	46.6	30	22.9	30	22.9	24	18.3
Yes	86	41	47.7	29	33.7	7	8.1	14	16.3	6	7.0
*p* value		0.11		0.06		0.005		0.24		0.02	

*Hospital of tuberculosis diagnosis* ^b^											
Municipal level hospital	127	77	60.6	70	55.1	25	19.7	20	15.7	22	17.3
District level hospital	85	36	42.4	16	18.8	11	12.9	22	25.9	7	8.2
*p* value		0.003		<0.001		0.20		0.07		0.06	

*History of tuberculosis treatment*											
Newly diagnosed	80	37	46.3	29	36.3	16	20.0	17	21.3	14	17.5
Relapsed	89	47	52.8	37	41.6	10	11.2	15	16.9	9	10.1
Chronic	48	34	70.8	24	50.0	11	22.9	12	25.0	7	14.6
*p* value^a^		0.01		0.13		0.91		0.74		0.50	

SLDs, second-line drugs; Ofx, ofloxacin; Am, amikacin; Km, kanamycin; Cm, capreomycin; XDR-TB, extensively drug-resistant tuberculosis.

^a^Mantel-Haenszel chi-square test.

^b^Five patients diagnosed in general hospitals were excluded.

**Table 2:** Diagnostic delays among MDR-TB patients, by history of tuberculosis and drug resistance pattern.

Variables	Total delay in DST completion^a^		Delay in TB diagnosis		Health system delay in DST completion^b^	
	Median	IQR	Median	IQR	Median	IQR
*All patients*	136	88–235	33	19–56	92	50–192

*History of tuberculosis treatment*						
Newly diagnosed	123	90–175	32	17.5–51.5	84.5	63.5–120
Relapsed	123	80–195	32	18–59	83	41–133
Chronic	420.5	110.5–873.5	33	20.5–54	297	47.5–821.5
*p* value	<0.001		0.76		<0.001	

*Drug resistance pattern*						
Resistant to FLDs only	115	86–175	34	18–61	82	42–115
Resistant to FLDs and Ofx	173.5	90–440.5	37.5	18.5–64.5	111	37.5–339.5
Resistant to FLDs and injectable drugs^c^	149	124–356.5	28	20–50.5	113	90.5–247.5
XDR-TB	148	81–221	25	18–41	104	58–191
*p* value	0.008		0.20		0.02	

DST, drug susceptibility testing; IQR, inter quarter range; FLDs, first-line drugs; Ofx, ofloxacin; XDR-TB, extensively drug-resistant tuberculosis.

*p* value: Kruskal-Wallis test.

^a^The duration from onset of TB symptoms in the most recent disease episode to the date of reporting DST results.

^b^The duration from TB diagnosis to the date of reporting DST results.

^c^Including amikacin, kanamycin, and capreomycin.

**Table 3:** Multivariate analysis of resistance to second-line drugs in longer/shorter diagnostic delays.

Diagnostic delays (days)	Adjusted OR (95% CI) for a delay longer than median days				
	Resistance to SLDs	Resistance to Ofx	Resistance to Am/Km	Resistance to Cm	XDR-TB
*Total delay in DST completion* (>136 versus ≤136)	2.22 (1.19–4.11)^*∗*^	2.03 (1.05–3.93)^*∗*^	1.13 (0.52–2.47)	1.39 (0.66–2.91)	1.22 (0.53–2.84)

*Delay in TB diagnosis* (>33 versus ≤33)	0.84 (0.47–1.51)	0.85 (0.45–1.58)	0.56 (0.26–1.21)	0.66 (0.33–1.35)	0.52 (0.22–1.21)

*Health system delay in DST completion* (>92 versus ≤92)	3.44 (1.79–6.61)^*∗∗*^	2.59 (1.32–5.09)^*∗*^	1.18 (0.54–2.55)	1.65 (0.79–3.45)	1.31 (0.57–3.03)

OR, odds ratio; CI, confidence interval; SLDs, second-line drugs; Ofx, ofloxacin; Am, amikacin; Km, kanamycin; Cm, capreomycin; XDR-TB, extensively drug-resistant tuberculosis; DST, drug susceptibility testing.

ORs were adjusted for age at diagnosis (continuous), sex, residence, level of TB hospital, and previous TB history.

^*∗*^ *p* < 0.05.

^*∗∗*^ *p* < 0.001.

**Table 4:** Multivariate analysis of resistance to second-line drugs in longer/shorter total and health system delay in DST completion, stratified by residence and TB history.

Variables	Adjusted OR (95% CI) for a delay longer than median days				
	Resistance to SLDs	Resistance to Ofx	Resistance to Am/Km	Resistance to Cm	XDR-TB
	*Total delay in DST completion*				

*Residence*					
Local	2.35 (1.08–5.11)^*∗*^	2.15 (0.97–4.76)	1.29 (0.53–3.14)	1.46 (0.59–3.63)	1.50 (0.57–3.97)
Domestic migrant	2.31 (0.79–6.81)	1.81 (0.52–6.36)	0.82 (0.14–4.71)	1.38 (0.36–5.34)	0.64 (0.10–4.16)

*TB history*					
Newly diagnosed	1.51 (0.54–4.23)	1.00 (0.33–3.08)	0.88 (0.26–3.01)	1.21 (0.39–3.82)	0.83 (0.23–3.01)
Relapsed	1.81 (0.69–4.73)	2.78 (0.88–8.82)	1.71 (0.38–7.69)	1.21 (0.32–4.49)	2.46 (0.50–12.17)
Chronic	7.14 (1.46–35.00)^*∗*^	4.65 (1.03–21.08)^*∗*^	1.27 (0.25–6.45)	2.75 (0.26–29.45)	1.39 (0.20–9.88)

	*Health system delay in DST completion*				

*Residence*					
Local	3.93 (1.71–9.07)^*∗*^	2.93 (1.28–6.70)^*∗*^	1.24 (0.52–3.00)	1.70 (0.68–4.26)	1.50 (0.58–3.91)
Domestic migrant	2.64 (0.91–7.63)	2.19 (0.65–7.42)	0.94 (0.17–5.31)	1.47 (0.38–5.78)	0.80 (0.13–4.97)

*TB history*					
Newly diagnosed	1.61 (0.56–4.63)	1.38 (0.44–4.33)	0.86 (0.25–3.01)	0.83 (0.26–2.71)	0.80 (0.21–3.01)
Relapsed	5.75 (1.89–17.52)^*∗*^	5.01 (1.39–18.05)^*∗*^	2.10 (0.45–9.76)	3.39 (0.81–14.16)	3.70 (0.65–21.10)
Chronic	5.09 (1.01–25.70)^*∗*^	4.95 (1.12–21.82)^*∗*^	1.38 (0.29–6.64)	2.18 (0.23–20.92)	1.51 (0.23–9.88)

OR, odds ratio; CI, confidence interval; SLDs, second-line drugs; Ofx, ofloxacin; Am, amikacin; Km, kanamycin; Cm, capreomycin; XDR-TB, extensively drug-resistant tuberculosis; DST, drug susceptibility testing.

ORs were adjusted for age at diagnosis (continuous), sex, and level of TB hospital.

^*∗*^ *p* < 0.05.

## References

[1] World Health Organization (WHO) (2013). *Definitions and Reporting Framework for Tuberculosis—2013 Revision*.

[2] Bastos M. L., Hussain H., Weyer K. (2014). Treatment outcomes of patients with multidrug-resistant and extensively drug-resistant tuberculosis according to drug susceptibility testing to first- and second-line drugs: an individual patient data meta-analysis. *Clinical Infectious Diseases*.

[3] World Health Organization (WHO) (2011). *Guidelines for the Programmatic Management of Drug-Resistant Tuberculosis—2011 Update*.

[4] Gandhi N. R., Nunn P., Dheda K. (2010). Multidrug-resistant and extensively drug-resistant tuberculosis: a threat to global control of tuberculosis. *The Lancet*.

[5] Dheda K., Gumbo T., Gandhi N. R. (2014). Global control of tuberculosis: from extensively drug-resistant to untreatable tuberculosis. *The Lancet Respiratory Medicine*.

[6] Yu M.-C., Wu M.-H., Jou R. (2008). Extensively drug-resistant tuberculosis, Taiwan. *Emerging Infectious Diseases*.

[7] Streicher E. M., Müller B., Chihota V. (2012). Emergence and treatment of multidrug resistant (MDR) and extensively drug-resistant (XDR) tuberculosis in South Africa. *Infection, Genetics and Evolution*.

[8] World Health Organization (WHO) (2015). *Global Tuberculosis Report 2015*.

[9] Becerra M. C., Appleton S. C., Franke M. F. (2011). Tuberculosis burden in households of patients with multidrug-resistant and extensively drug-resistant tuberculosis: a retrospective cohort study. *The Lancet*.

[10] Zhao Y., Xu S., Wang L. (2012). National survey of drug-resistant tuberculosis in China. *The New England Journal of Medicine*.

[11] Li J., Zhang Y. Y., Gui X. H. (2012). Prevalence and risk factors on the resistance related to second-line drugs among multi-drug resistant tuberculosis cases in Shanghai, China. *Chinese Journal of Epidemiology*.

[12] Xu P., Li X., Zhao M. (2009). Prevalence of fluoroquinolone resistance among tuberculosis patients in Shanghai, China. *Antimicrobial Agents and Chemotherapy*.

[13] Zhang X., Yin J., Li H. (2015). Diagnostic and treatment delays of multidrug-resistant tuberculosis before initiating treatment: a cross-sectional study. *Tropical Medicine & International Health*.

[14] World Health Organization (2009). *Guidelines for Surveillance of Drug Resistance in Tuberculosis*.

[15] World Health Organization (2008). *Policy Guidance on Drug-Susceptibility Testing (DST) of Second-Line Antituberculosis Drugs*.

[16] World Health Organization (2010). *Multidrug and Extensively Drug-Resistant TB (M/XDR-TB): 2010 Global Report on Surveillance and Response*.

[17] World Health Organization (2009). *Treatment of Tuberculosis: Guidelines for National Programmes*.

[18] Wang Y. *Prevention and Treatment of Multidrug-Resistant Tuberculosis*.

[19] Falzon D., Gandhi N., Migliori G. B. (2013). Resistance to fluoroquinolones and second-line injectable drugs: impact on multidrug-resistant TB outcomes. *European Respiratory Journal*.

[20] Meyssonnier V., Li X., Shen X. (2013). Factors associated with delayed tuberculosis diagnosis in China. *European Journal of Public Health*.

[21] Sreeramareddy C. T., Panduru K. V., Menten J., Van den Ende J. (2009). Time delays in diagnosis of pulmonary tuberculosis: a systematic review of literature. *BMC Infectious Diseases*.

[22] Singla R., Sarin R., Khalid U. K. (2009). Seven-year DOTS-Plus pilot experience in India: results, constraints and issues. *International Journal of Tuberculosis and Lung Disease*.

[23] Paramasivan C. N., Rehman F., Wares F. (2010). First- and second-line drug resistance patterns among previously treated tuberculosis patients in India. *International Journal of Tuberculosis and Lung Disease*.

[24] Smith S. E., Ershova J., Vlasova N. (2015). Risk factors for acquisition of drug resistance during multidrug-resistant tuberculosis treatment, Arkhangelsk Oblast, Russia, 2005–2010. *Emerging Infectious Diseases*.

[25] Bakuła Z., Napiórkowska A., Kamiński M. (2016). Second-line anti-tuberculosis drug resistance and its genetic determinants in multidrug-resistant *Mycobacterium tuberculosis* clinical isolates. *Journal of Microbiology, Immunology and Infection*.

[26] Hu Y., Mathema B., Zhao Q. (2015). Acquisition of second-line drug resistance and extensive drug resistance during recent transmission of *Mycobacterium tuberculosis* in rural China. *Clinical Microbiology and Infection*.

[27] Calver A. D., Falmer A. A., Murray M. (2010). Emergence of increased resistance and extensively drug-resistant tuberculosis despite treatment adherence, South Africa. *Emerging Infectious Diseases*.

[28] Li X., Zhang Y., Shen X. (2007). Transmission of drug-resistant tuberculosis among treated patients in Shanghai, China. *Journal of Infectious Diseases*.

[29] Cox H. S., Sibilia C., Kalon S. (2008). Emergence of extensive drug resistance during treatment for multidrug-resistant tuberculosis. *The New England Journal of Medicine*.

[30] Escombe A. R., Oeser C. C., Gilman R. H. (2007). Natural ventilation for the prevention of airborne contagion. *PLoS Medicine*.

[31] Wang F., Shao L., Fan X. (2015). Evolution and transmission patterns of extensively drug-resistant tuberculosis in China. *Antimicrobial Agents and Chemotherapy*.

[32] Ioerger T. R., Feng Y., Chen X. (2010). The non-clonality of drug resistance in Beijing-genotype isolates of *Mycobacterium tuberculosis* from the Western Cape of South Africa. *BMC Genomics*.

[33] Porwal C., Kaushik A., Makkar N. (2013). Incidence and risk factors for extensively drug-resistant tuberculosis in Delhi Region. *PLoS ONE*.

[34] Dooley K. E., Golub J., Goes F. S., Merz W. G., Sterling T. R. (2002). Empiric treatment of community-acquired pneumonia with fluoroquinolones, and delays in the treatment of tuberculosis. *Clinical Infectious Diseases*.

[35] Chen T.-C., Lu P.-L., Lin C.-Y., Lin W.-R., Chen Y.-H. (2011). Fluoroquinolones are associated with delayed treatment and resistance in tuberculosis: a systematic review and meta-analysis. *International Journal of Infectious Diseases*.

[36] Jeon C. Y., Calver A. D., Victor T. C., Warren R. M., Shin S. S., Murray M. B. (2011). Use of fluoroquinolone antibiotics leads to tuberculosis treatment delay in a South African gold mining community. *International Journal of Tuberculosis and Lung Disease*.

[37] Devasia R. A., Blackman A., Gebretsadik T. (2009). Fluoroquinolone resistance in *Mycobacterium tuberculosis*: the effect of duration and timing of fluoroquinolone exposure. *American Journal of Respiratory and Critical Care Medicine*.

[38] Hillemann D., Rüsch-Gerdes S., Richter E. (2009). Feasibility of the GenoType MTBDRsl assay for fluoroquinolone, amikacin-capreomycin, and ethambutol resistance testing of *Mycobacterium tuberculosis* strains and clinical specimens. *Journal of Clinical Microbiology*.

